# Association of Glucagon to Insulin Ratio and Metabolic Syndrome in Patients with Type 2 Diabetes

**DOI:** 10.3390/jcm12185806

**Published:** 2023-09-06

**Authors:** Jisun Bang, Sang Ah Lee, Gwanpyo Koh, Soyeon Yoo

**Affiliations:** 1 Department of Internal Medicine, Jeju National University Hospital, Jeju 63241, Republic of Korea; 2 Department of Internal Medicine, Jeju National University College of Medicine, Jeju 63241, Republic of Korea

**Keywords:** type 2 diabetes mellitus, metabolic syndrome, glucagon, insulin, cardiovascular risk

## Abstract

There is a growing interest in the role of glucagon in type 2 diabetes mellitus (T2DM). Glucagon and insulin regulate glucose and lipid metabolism. Metabolic syndrome is an important risk factor for cardiovascular disease in patients with T2DM. We investigated the association between glucagon to insulin ratio and metabolic syndrome in patients with T2DM. This is a cross-sectional study involving 317 people with type 2 diabetes. Glucagon and insulin levels were measured in a fasted state and 30 min after ingesting a standard mixed meal. The Criteria of the International Diabetes Federation defined metabolic syndrome. Two hundred nineteen (69%) of the subjects had metabolic syndrome. The fasting glucagon to insulin ratio was significantly lower in patients with metabolic syndrome (14.0 ± 9.7 vs. 17.3 ± 10.3, *p* < 0.05). The fasting glucagon to insulin ratio was significantly lowered as the number of metabolic syndrome components increased. In hierarchical logistic regression analysis, the fasting glucagon to insulin ratio significantly contributed to metabolic syndrome even after adjusting for other covariates. The fasting glucagon to insulin ratio is inversely associated with metabolic syndrome in patients with type 2 diabetes. This suggests that glucagon-targeted therapeutics may reduce cardiovascular risk by improving metabolic syndrome.

## 1. Introduction

About 537 million individuals aged 20 to 79 have diabetes mellitus (DM), making up 10.5% of the worldwide population in that age range. By 2030, the number of patients with DM is expected to hit 643 million (11.3%), increasing to 783 million (12.2%) by 2045. Around 240 million worldwide remain unaware they are diabetic, and nearly 90% of these undiagnosed cases are in lower- to middle-income regions [1]. DM is increasingly prevalent worldwide, and the consequent socioeconomic burden is continuously rising. Recent research on the etiology of type 2 diabetes mellitus (T2DM) has identified various mechanisms that contribute to hyperglycemia, in addition to insulin resistance and beta-cell dysfunction. As a result, the development of drugs targeting these mechanisms is actively underway. Glucagon is a hormone secreted by alpha cells in the pancreas in response to low blood glucose levels. It raises blood glucose levels by stimulating glycogenolysis and glucose synthesis and inhibiting glycogenesis in the liver [2]. In patients with T2DM, hyperglucagonemia due to inappropriately increased alpha cells function has been reported to contribute to hyperglycemia by stimulating glucose synthesis in the liver [3]. Recent studies have observed higher fasting blood glucagon concentrations, loss of glucose-stimulated inhibition of glucagon secretion, and disruption of postprandial insulin-glucagon interactions in patients with T2DM compared to patients without DM [4]. However, research on the underlying mechanisms and clinical implications of glucagon dysregulation in T2DM remains insufficient.

In addition to glucose metabolism, glucagon is also involved in lipid metabolism, fatty acid oxidation, ketone body production, energy consumption, and appetite regulation [5]. The secretion and action of glucagon are heavily affected by insulin secretion. Thus, the glucagon to insulin ratio is a more appropriate parameter than blood glucagon concentration when analyzing the effects of glucagon [6].

The presence of a cluster of clinically significant cardiovascular risk factors in an individual is referred to as metabolic syndrome, and patients with metabolic syndrome have higher cardiovascular disease incidence and mortality rates compared to the general population [7]. A substantial number of patients with DM (70–80%) have metabolic syndrome, and the increase in the incidence of cardiovascular disease in these patients is higher than that in non-diabetic individuals with metabolic syndrome [8]. Thus, analyzing the association between the glucagon to insulin ratio and metabolic syndrome in patients with T2DM would indirectly shed light on the effect of glucagon on cardiovascular disease.

Accordingly, our aim was to investigate the relationship between the glucagon to insulin ratio and metabolic syndrome in T2DM patients, in order to identify the effect of glucagon on cardiovascular disease.

## 2. Materials and Methods

### 2.1. The Study Population

From the data and serum samples of patients with DM collected by Endocrinology and Metabolism at Jeju National University Hospital, a database was established for diabetes research in the Korean population [IRB No. JEJUNUH 2010-33]); patients who satisfy the following criteria were included. Inclusion criteria were as follows: (1) aged 20–75 years, and (2) patients who underwent mixed meal tolerance test (MMTT). Patients with stage 4 or 5 chronic kidney disease (estimation of the glomerular filtration rate [eGFR] < 30 mL/min), liver cirrhosis with ascites, or infectious, inflammatory diseases were excluded. Additionally, patients who have used dipeptidyl peptidase-4 (DPP-4) inhibitors, Glucagon-like peptide 1 (GLP-1) receptor antagonists, or insulin injections, which may affect glucagon and incretin concentrations, were excluded.

### 2.2. Method

Following an overnight fast of at least eight hours, blood samples were collected to measure glucose, glycated hemoglobin (HbA1c), C-peptide, insulin, glucagon, intact GLP-1(iGLP-1), intact glucose-dependent insulinotropic polypeptide (iGIP), total cholesterol, triglycerides, high-density lipoprotein cholesterol (HDL-C), low-density lipoprotein cholesterol (LDL-C), aspartate aminotransferase (AST), alanine aminotransferase (ALT), high-sensitivity C-reactive protein (hs CRP), fibrinogen, uric acid, blood urea nitrogen (BUN), and creatinine (Cr). Early morning spot urine sample was obtained to measure urinary albumin-creatinine ratio (mg/dl) (UACR). Subsequently, the participants were provided standard mixed meals (480 kcal, carbohydrate: protein: fat = 2.8:1:1). The standard mixed meals consisted of rice, soup, three side dishes, and kimchi, with consistent nutrient ratios across meals. The subjects were asked to complete their meal within 20 min. Additional blood samples were collected 30 min after the meal’s start to measure glucose, C-peptide, insulin, glucagon, iGLP-1, and iGIP. iGLP-1 and iGIP levels were measured from blood samples stored in chilled EDTA tubes, to which aprotinin (250 KIU/mL blood; Sigma-Aldrich, St Louis, MO, USA) and a DPP-4 inhibitor (10 μL/mL blood; Merck Millipore, Darmstadt, Germany) had been added.

Age, DM duration, comorbidity, and current use of antidiabetic medications were collected from the electronic medical records at Jeju National University. Height and weight were measured using an electronic scale. Systolic and diastolic blood pressure (BP) was measured after 10 min of rest, and the average of two measurements was used. Waist circumference (WC) was measured at the midpoint between the bottom of the ribs and the iliac crest, with the participant standing straight with legs shoulder-width apart.

Metabolic syndrome was diagnosed in accordance with the International Diabetes Federation criteria [9]:

(1) WC ≥ 90 cm (male), ≥85 cm (female);
(2) BP ≥ 130/85 mmHg or current use of antihypertensive medications;
(3) Fasting glucose ≥ 100;
(4) Triglyceride ≥ 150 mg/dL;
(5) HDL-C < 40 mg/dL (male), <50 mg/dL (female).

Since this study was conducted on patients with T2DM, all patients met the fasting glucose criterion, and those who additionally met at least two other diagnostic criteria were considered to have metabolic syndrome. 

### 2.3. Measurements of Biochemical Markers

Plasma glucose levels were measured by the glucose oxidase method using the TBA-200FR chemical analyzer (Toshiba, Tokyo, Japan). HbA1c levels were measured by ion-exchange high-performance liquid chromatography using HLC-723G8 (Tosoh, South San Francisco, CA, USA). C-peptide levels were measured using Modular Analytics E170 electrochemiluminescence immunoassays (Hitachi, Tokyo, Japan). Creatinine and ALT levels were measured using the TBA-200FR chemical analyzer, while eGFR was calculated using the Modification of Diet in Renal Disease equation. For the quantification of iGLP-1 (GLP-1 (7–36) amide, GLP-1 (7–37)) and iGIP (GIP (1–42)), a measurement kit based on the principle of sandwich enzyme immunoassay (Code Nos. 27784 and 27201, respectively; Immuno-Biological Laboratories Co. Ltd., Gunma, Japan) was purchased and used. For the quantification of glucagon, a glucagon chemiluminescent kit based on sandwich enzyme-linked immunosorbent assay (Cat. No. EZGLU-30K, Merck KGaA, Darmstadt, Germany) was purchased and used.

### 2.4. Statistical Analysis

Results for continuous variables were presented as mean ± standard error, and those for categorical variables were presented as percentages. To examine the characteristics of patients with T2DM and metabolic syndrome, participants were divided into the metabolic syndrome and non-metabolic syndrome group. The means of continuous variables were compared using student’s *t*-tests, and the means of categorical variables were compared with chi-squared tests. To examine the relationship between the glucagon to insulin ratio and metabolic syndrome, we conducted an analysis of variance (ANOVA) coupled with a linear trend test. Patients were categorized into five groups based on the number of metabolic syndrome components they exhibited. Hierarchical logistic regression was performed to comprehensively analyze the effects of various factors on metabolic syndrome. Model 1 included age, sex, and fasting glucagon to insulin ratio. In Model 2, LDL-C, which exhibited a significant difference between the metabolic syndrome and non-metabolic syndrome groups, along with factors that could influence it such as the use of lipid-lowering agents, were added. Additionally, HbA1C, which reflects the degree of blood glucose control, was included. The final model (Model 3) incorporated fasting iGLP1 and fasting iGIP, which are incretins potentially influencing glucagon/insulin secretion, along with well-established cardiovascular risk factors such as hs CRP, fibrinogen, uric acid, and ALT. All analyses were conducted using the SPSS 18.0 (SPSS Inc., Chicago, IL, USA), and *p* < 0.05 was deemed statistically significant.

## 3. Results

In this study, 317 individuals were involved, comprising 205 men (64.7%) and 112 women (35.3%), with an average age of 59.1 ± 11.7 years. The average BMI was 25.9 ± 3.7 kg/m^2^. Men had an average waist circumference of 90.5 ± 8.5 cm and women, 91.1 ± 10.9 cm, hinting at prevalent abdominal obesity in both genders. The average diabetes duration was 9.4 ± 8.3 years, and the mean HbA1c was 8.7 ± 2.2%, pointing to suboptimal blood glucose management in many patients.

**Figure 1:**
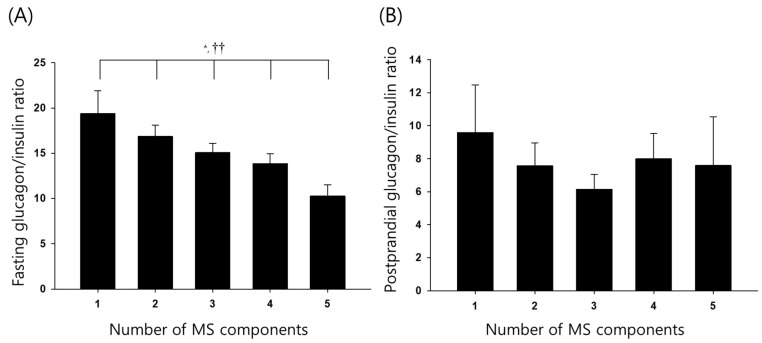
Differences in fasting glucagon/insulin ratio (**A**) and postprandial glucagon/insulin ratio (**B**) according to the number of components of metabolic syndrome. Values are expressed as mean ± SE. MS, metabolic syndrome; ANOVA, analysis of variance; *p* for trend, *p* value by the test for linear trend. * *p* ANOVA < 0.05; †† *p* for trend < 0.01.

From a total of 317 study participants, 219 participants (69%) had metabolic syndrome. Regarding metabolic syndrome components, the metabolic syndrome group had significantly higher WC, BP, and triglyceride and significantly lower HDL-C. It is not surprising that these show significant differences between the two groups as they are criteria for diagnosing metabolic syndrome. The metabolic syndrome and non-metabolic syndrome groups also significantly differed in body mass index (BMI), baseline C-peptide, and LDL-C. Fasting and postprandial glucagon concentrations did not significantly differ between the two groups, but the fasting glucagon to insulin ratio was significantly lower in the metabolic syndrome group (Table 1). The participants were divided into five groups based on the number of metabolic syndrome components they have, and they were compared using ANOVA with the linear trend test. The fasting glucagon to insulin ratio significantly decreased with increasing metabolic syndrome components (Figure 1). The effects of the glucagon to insulin ratio on the presence of metabolic syndrome among patients with DM were analyzed using hierarchical logistic regression. Model 1 analyzed the effects of age, sex, and glucagon to insulin ratio. The glucagon to insulin ratio was identified as a significant predictor of metabolic syndrome. The glucagon to insulin ratio was a significant predictor even after adjusting for HbA1c, LDL-C, and use of hyperlipidemia medications, and the same was true after additionally adjusting for incretins (iGLP-1, iGIP), hsCRP (cardiovascular risk factor), fibrinogen, and uric acid (Table 2). 

## 4. Discussion

Metabolic syndrome increases the risk for cardiovascular disease in patients with T2DM. In the present study, the glucagon to insulin ratio was significantly lower in patients with T2DM who also have metabolic syndrome. The glucagon to insulin ratio was a significant predictor of metabolic syndrome even after adjusting for confounders through multiple regression analysis.

Bonora et al. [7] conducted a cohort study to assess the cardiovascular disease risk among T2DM subjects with metabolic syndrome, based on WHO criteria. Using a sample from the Verona Diabetes Complications Study, they found that 92.3% of the subjects had metabolic syndrome. These individuals exhibited a higher baseline cardiovascular disease prevalence of 32.9% compared to 17.8% in others. During a 4.5-year follow-up, the group with metabolic syndrome experienced a notably higher rate of cardiovascular disease events (19.9% vs. 3.9%). When accounting for other factors, metabolic syndrome independently predicted an almost five-fold increase in cardiovascular disease risk. About 70% of the T2DM patients who participated in this study had metabolic syndrome. Bonora and colleagues diagnosed metabolic syndrome based on WHO criteria, whereas our study used the International Diabetes Federation criteria for diagnosis. This could account for the difference in prevalence rates.

Insulin and glucagon have a reciprocal relationship, with opposing functions that modulate each other’s secretion. As such, the glucagon to insulin ratio is a better parameter than the absolute values of insulin or glucagon to be used when analyzing carbohydrate and lipid metabolisms and their actions [10].

Sodium-glucose cotransporter-2 (SGLT-2) inhibitors, which are oral hypoglycemic agents, primarily lower blood glucose by increasing the excretion of glucose in the urine. However, cardiovascular outcomes studies of SGLT-2 inhibitors have shown a reduction in cardiovascular complications and mortality rates in patients with T2DM [11]. The reduction in cardiovascular complications and mortality rates associated with SGLT-2 inhibitors primarily stemmed from reduced hospitalizations and deaths from heart failure. Subsequently, further studies were conducted to specifically investigate the effects of SGLT-2 inhibitors on heart failure. In the DAPA-HF Trial [12], involving 4744 heart failure patients with a reduced ejection fraction, dapagliflozin (an SGLT-2 inhibitor) was compared to a placebo over an average of 18.2 months. The study found that dapagliflozin reduced the risk of worsening heart failure or cardiovascular death by 26% compared to placebo. This benefit was consistent irrespective of whether the patients had T2DM or not. Adverse event rates were similar across the two groups. In the EMPEROR-Reduced Trial [13], of 3730 heart failure patients with a reduced ejection fraction (≤40%), the use of empagliflozin, an SGLT-2 inhibitor, was compared with a placebo over a median duration of 16 months. Empagliflozin resulted in a 25% reduction in the risk of cardiovascular death or heart failure hospitalization compared to the placebo, a benefit observed regardless of the diabetes status of the patients. Based on these large-scale clinical trials, which included patients without DM, it has been substantiated that SGLT-2 inhibitors reduce hospitalizations and deaths from heart failure. Consequently, SGLT-2 inhibitors are now recommended as a therapeutic agent for heart failure in the presence or absence of T2DM. 

The mechanism by which SGLT-2 inhibitors improve heart failure is not fully understood. Several theories have been proposed, including metabolic, diuretic, and pleiotropic off-target effects. SGLT-2 inhibitors directly stimulate alpha cells, increasing glucagon secretion [14]. Additionally, the increase in glucose excretion in urine reduces insulin secretion, thus increasing the glucagon to insulin ratio. As a result, lipolysis is stimulated, and ketone body production increases. Recently, it has been reported that the use of Empagliflozin in patients with T2DM leads to a decrease in serum insulin, an increase in glucagon, and an increase in serum ketone bodies [15]. Regarding ketone bodies, the “Super-fuel” hypothesis has been proposed, which suggests that serum ketone bodies can serve as an effective energy source for the myocardium, contributing to improved cardiac function in patients with T2DM [16]. Murashige et al. found that the failing heart had increased consumption of ketones and lactate and exhibited elevated rates of proteolysis [17]. In this quantitative study evaluating the arteriovenous gradient for metabolites, no significant glucose extraction by the non-failing human heart was noted. Calculations indicated that around 85% of the heart’s ATP was generated from free or lipoprotein-associated fatty acids. Ketones contributed 6.4%, amino acids 4.6%, lactate 2.8%, and acetate 2%. For patients with heart failure (with a left ventricular ejection fraction of less than 40%), the ATP derived from ketones almost tripled to 16.4%, while that from lactate nearly doubled to 5.0% [17]. Recent research has identified a connection between mitochondrial hyperacetylation and inflammation in the pathogenesis of heart failure with preserved ejection fraction, suggesting that elevating β-hydroxybutyrate levels might present a potential therapeutic strategy [18]. This study delved into the metabolic mechanisms behind heart failure with preserved ejection fraction using a novel animal model that involved aged mice, high-fat diets, and desoxycorticosterone pivalate. Mice with heart failure with preserved ejection fraction displayed both mitochondrial hyperacetylation and inflammation. However, by increasing β-hydroxybutyrate levels, inflammation and mitochondrial dysfunction were notably reduced. There are multiple reasons for ketosis in diabetic patients. However, it is known that initiating ketogenesis begins with a shift in the molar ratio of glucagon to insulin in plasma such that there is a relative or absolute excess of glucagon and a deficiency of insulin [19]. In our study, individuals with metabolic syndrome had a significantly lower glucagon to insulin ratio. Furthermore, the glucagon to insulin ratio dropped considerably with the rise in the number of metabolic syndrome elements. 

In patients with T2DM, an elevated glucagon to insulin ratio may lead to increased blood glucose levels. This observation aligns with findings from previous studies. Lee et al. [20] explored the role of α-cell dysfunction and its relationship with glucagon excess in T2DM. In their study involving 451 non-insulin-treated patients, those with higher glucagon to insulin ratios exhibited elevated HbA1c levels. A notable correlation was observed between HbA1c levels and both fasting and post-meal glucagon to insulin ratios. Significantly, those with higher post-meal ratios faced a greater risk of uncontrolled blood sugar, suggesting that an imbalanced high glucagon to insulin ratio could impair glucose control in type 2 diabetes patients. However, it is essential to consider that glucagon impacts both lipid and glucose metabolism. An increased glucagon to insulin ratio might promote fatty liver by reducing lipid metabolism, especially hepatic lipogenesis. Glucagon has been demonstrated to inhibit lipogenesis and reduce the secretion of triglycerides and very low-density lipoproteins in hepatocytes [21,22]. Furthermore, glucagon boosts the transcription factor cAMP responsive element binding (CREB) protein in hepatocytes, which in turn enhances the transcription of carnitine acyl transferase 1 (CPT-1) [23]. Beta-oxidation increases as a result, and these effects of glucagon are suppressed by insulin. Hence, it has been reported that the metabolic state of hepatocytes is primarily dictated by the glucagon to insulin ratio rather than the individual concentration of these hormones [24].

Fibroblast growth factor 21 (FGF21) is a hormone produced by various tissues secreted in the fasting state and involved in metabolic responses to fasting [25]. FGF21 and FGF21 analogs have been shown to promote weight loss and reduction in fatty liver in numerous preclinical studies [26]. In a study on ten healthy adults, the glucagon to insulin ratio was the most important regulator of splanchnic FGF21 secretion [27]. Glucagon receptor antagonist agents, developed for the treatment of DM, have been found to significantly lower blood glucose concentration in T2DM. Still, the use of these agents has been associated with increased hepatic fat fraction, body weight, and total cholesterol [28]. Recently developed glucagon and GLP-1 co-agonists effectively lower blood glucose and ameliorate fatty liver and lipid metabolism, and relevant research is underway. Non-alcoholic fatty liver is a prevalent comorbidity in patients with metabolic syndrome, and several epidemiology studies have demonstrated an association between the two conditions [29]. In a cohort study of 11,647 participants, the relationship between metabolic syndrome and the prevalence and severity of non-alcoholic fatty liver disease was examined [30]. The overall prevalence of non-alcoholic fatty liver disease was 18.2% (95% CI 16.5–19.9). Among individuals with metabolic syndrome, this prevalence was substantially higher at 43.2%. Notably, for those meeting all five components of the metabolic syndrome criteria, the prevalence surged to 67%. Among those with moderate to severe steatosis, 6.6% showed signs of advanced hepatic fibrosis. This percentage nearly doubled for individuals with metabolic syndrome, reaching 30% for those meeting all five metabolic syndrome criteria [30]. These findings indicate a robust association between metabolic syndrome and non-alcoholic fatty liver. As the components of the metabolic syndrome increase, there is a corresponding rise in both the prevalence of non-alcoholic fatty liver and the risk of hepatic fibrosis. In this investigation, a higher number of metabolic syndrome components correlated with an elevated glucagon to insulin ratio. Interestingly, this ratio was lower in patients with T2DM combined with metabolic syndrome. In a cross-sectional study of 172 participants with T2DM [31], the relationship between the glucagon to insulin ratio and the presence of non-alcoholic fatty liver disease was examined. Participants were categorized into tertiles based on their fasting and postprandial glucagon to insulin ratios and were assessed for non-alcoholic fatty liver via ultrasonography. The results showed a significant decrease in the prevalence of non-alcoholic fatty liver across the tertiles for both fasting and postprandial glucagon to insulin ratios [31].

This study has a few limitations. First, this study’s retrospective design is an inherent limitation, as it restricts the ability to establish cause-and-effect relationships. Additionally, there was an unequal distribution of participants between the groups with and without metabolic syndrome. Thus, we attempted to conduct a more precise analysis through logistic regression in addition to simple intergroup comparisons. Second, we did not directly analyze the risk for cardiovascular disease. We could not analyze the incidence of cardiovascular events due to the cross-sectional nature of this study. Instead, we analyzed the relationship between metabolic syndrome and glucagon to insulin ratio, which are strongly associated with cardiovascular disease in patients with T2DM. Third, we did not measure blood ketone body concentrations. Based on the glucagon to insulin ratio, we can infer that the blood ketone body concentrations would differ, but no test was performed at the time of data collection. Subsequent studies should perform more accurate comparisons.

In this study, the metabolic syndrome group had a significantly lower glucagon to insulin ratio, and the glucagon to insulin ratio significantly decreased with an increasing number of metabolic syndrome components. The glucagon to insulin ratio remained a predictor of metabolic syndrome even after adjusting for several factors. These findings suggest that cardiovascular risk increases with decreasing glucagon to insulin ratio in patients with T2DM. The mechanism may involve increased ketone body production and consequent improvement of cardiac functions with an increasing glucagon to insulin ratio. There has been a growing emphasis on the cardioprotective effects of SGLT-2 inhibitors in recent years, and GLP-1/GIP/glucagon triple agonist agents are being developed with promising expectations for their glycemic control and weight loss. Therefore, the use of these drugs should be actively considered in patients with metabolic syndrome.

## Figures and Tables

**Table 1:** Comparison of clinical and biochemical variables according to the presence of metabolic syndrome.

	Metabolic Syndrome (*n* = 219)	No Metabolic Syndrome (*n* = 98)	*p* Value
Age, years	58.5 ± 12.2	60.5 ± 10.3	0.175
Gender, male, %	63.8	67.0	0.594
Body mass index, kg/cm^2^	26.8 ± 3.7	23.7 ± 2.8	<0.001
Waist circumference, cm	93.6 ± 8.1	84.3 ± 9	<0.001
Systolic BP, mmHg	140.7 ± 16.9	131.2 ± 17.6	<0.001
DM duration, years	9.2 ± 8.4	9.8 ± 8.3	0.535
HbA1c, %	8.8 ± 2.2	8.8 ± 2.4	0.921
Fasting glucose, mg/dL	169.3 ± 60	163.4 ± 66.7	0.461
Basal C-peptide, ng/mL	2.4 ± 1.3	1.8 ± 0.8	<0.001
AST, U/L	28.6 ± 21.7	25.2 ± 16.4	0.209
ALT, U/L	35.2 ± 25.4	29.7 ± 31.4	0.130
eGFR, mL/min	68.9 ± 14.4	69 ± 13.3	0.970
hs CRP, mg/dL	0.37 ± 1.32	0.41 ± 1.47	0.794
Fibrinogen, mg/dL	305.71 ± 76.47	309.87 ± 75.76	0.673
Uric acid, mg/dL	5.14 ± 1.34	4.82 ± 1.51	0.082
Total cholesterol, mg/dL	174.4 ± 40.1	166.7 ± 33.4	0.116
Triglyceride, mg/dL	164.6 ± 95	89.5 ± 36.4	<0.001
HDL cholesterol, mg/dL	43.7 ± 11.5	53.9 ± 12.1	<0.001
LDL cholesterol, mg/dL	105.8 ± 35.7	95.4 ± 31.2	0.019
UACR, mg/g Cr	241.3 ± 595.3	197.4 ± 461.5	0.580
Lipid-lowering agent, %	45.9	52.3	0.319
Antidiabetic regimen			
Sulfonylurea, %	57.7	48.9	0.169
Metformin, %	73.5	67.0	0.268
Thiazolidinedione, %	5.1	8.0	0.349
α-Glucosidase inhibitor, %	4.6	8.0	0.256
Fasting insulin, mg/dL	8.9 ± 6.5	6.5 ± 5.0	0.001
Postprandial insulin, mg/dL	22.1 ± 15.6	16.7 ± 11.6	0.006
Fasting glucagon, ng/L	87.1 ± 37.8	82.8 ± 38	0.375
Postprandial glucagon, ng/L	88.1 ± 54	78.5 ± 49.5	0.155
Fasting Glucagon/Insulin ratio	14.0 ± 9.7	17.3 ± 10.3	0.010
Postprandial Glucagon/Insulin ratio	7.0 ± 9.9	7.9 ± 9.9	0.517
Fasting iGLP-1, pmol/L	5.6 ± 3.2	5.9 ± 4.8	0.590
Post prandial iGLP-1, pmol/L	10.9 ± 7.3	12.9 ± 13.3	0.180
Fasting iGIP, pmol/L	4.1 ± 4.2	3.4 ± 3.2	0.198
Post prandial iGIP, pmol/L	22.1 ± 6.6	21.3 ± 7.4	0.369

Data are expressed as mean ± standard deviation or frequencies (%). Student’s *t*-test or chi-square test was performed. BP, blood pressure; DM, diabetes mellitus; eGFR, estimation of the glomerular filtration rate; AST, aspartate aminotransferase; ALT, alanine aminotransferase; hs CRP, high-sensitivity C-reactive protein; HDL cholesterol, high-density lipoprotein cholesterol; LDL cholesterol, low-density lipoprotein cholesterol; UACR, urinary albumin to creatinine ratio; iGLP-1, intact glucagon-like peptide 1; iGIP, intact glucose-dependent insulinotropic polypeptide.

**Table 2:** Multivariate hierarchical logistic regression analysis predicting metabolic syndrome.

	Model 1	Model 2	Model 3
	OR (95% CI)	OR (95% CI)	OR (95% CI)
Age, years	0.99 (0.96–1.01)	0.99 (0.97–1.01)	0.99 (0.96–1.02)
Gender, male	0.88 (0.50–1.50)	0.85 (0.49–1.49)	1.25 (0.66–2.38)
Fasting glucagon/insulin ratio	0.97 (0.95–0.99)	0.97 (0.94–0.99)	0.97 (0.94–0.99)
HbA1C (%)		1.02 (0.90–1.15)	1.05 (0.92–1.21)
LDL cholesterol, mg/dL		1.01 (1.00–1.02)	1.01 (1.00–1.02)
Use of lipid-lowering agent		1.14 (0.63–2.03)	0.80 (0.44–1.48)
Fasting iGLP-1, pmol/L			0.96 (0.89–1.04)
Fasting iGIP, pmol/L			1.05 (0.96–1.15)
hs CRP, mg/dL			1.00 (0.80–1.26)
Fibrinogen, mg/dL			0.99 (0.99–1.00)
Uric acid, mg/dL			1.17 (0.94–1.46)
ALT, U/L			1.01 (1.00–1.02)
Cox and Snell R^2^	0.029	0.049	0.79
Nagelkerke R^2^	0.04	0.069	0.113
Model χ^2^	8.1 *	14.3 *	22.7 *

Metabolic syndrome was the dependent variable. OR, odds ratios; CI, confidence interval; LDL cholesterol, low-density lipoprotein cholesterol; iGLP-1, intact glucagon-like peptide 1; iGIP, intact glucose-dependent insulinotropic polypeptide; hs CRP, high-sensitivity C-reactive protein; ALT, alanine aminotransferase. * *p* < 0.05.

## Data Availability

Data can be obtained from the corresponding author.
